# COVID-19 Pandemic Stress-Induced Somatization in Transplant Waiting List Patients

**DOI:** 10.3389/fpsyt.2021.671383

**Published:** 2021-07-06

**Authors:** Jolana Wagner-Skacel, Nina Dalkner, Susanne Bengesser, Michaela Ratzenhofer, Nadja Fink, Judith Kahn, Rene Pilz, Sabrina Mörkl, Melanie Lenger, Christian Fazekas, Franziska Matzer, Mary Butler, Eva Z. Reininghaus, Helmut Müller, Daniela Kniepeiss

**Affiliations:** ^1^Department of Medical Psychology and Psychotherapy, Medical University of Graz, Graz, Austria; ^2^Department of Psychiatry and Psychotherapeutic Medicine, Medical University of Graz, Graz, Austria; ^3^General, Visceral and Transplant Surgery, Medical University of Graz, Graz, Austria; ^4^Transplant Center Graz, Graz, Austria; ^5^Department of Psychiatry, University College Cork, Cork, Ireland

**Keywords:** COVID-19, somatization, depression, patient education, mental health, SARS-CoV-2, transplant waiting list

## Abstract

**Background:** The coronavirus disease 2019 (COVID-19) pandemic has resulted in widespread socioeconomic restrictions including quarantine, social distancing and self-isolation. This is the first study investigating the psychological impact of the pandemic on patients waiting for liver or kidney transplantation, a particularly vulnerable group.

**Methods:** Twenty-seven patients on the transplantation waiting list and 43 healthy controls took part in an online survey including the Beck Depression Inventory (BDI-2), the Brief Symptom Inventory-18 (BSI-18), the Pittsburgh Sleep Quality Index (PSQI), the Alcohol Use Identification Test (AUDIT-C), the 12-item Operationalized Psychodynamic Diagnosis Structure Questionnaire (OPD-SQS) and a questionnaire to determine cognitions and beliefs, attitude and fear related to COVID-19.

**Results:** BSI-18 Somatization was increased in waiting list patients compared to controls. Correlation analyses indicated a relationship between Somatization and the fear of contracting the coronavirus in the patient group; however this association was weak. In patients and controls, other psychologicial symptoms (depression, anxiety) correlated highly with emotional distress due to social distancing. There were no differences between patients and controls in depression scores and sleep disturbances. Alcohol consumption and personality structure were not related to COVID-19 fears.

**Conclusion:** In times of the first lockdown during the COVID-19 pandemic, patients on the transplantation waiting list have high somatization symptoms associated with COVID-19 fears. As vulnerable group, they need psychological counseling to improve mental well-being during times of crisis.

## Introduction

The coronavirus disease 2019 (COVID-19) pandemic has changed the lives of everybody and the whole world has experienced a state of emergency (1, 2). The implementation of strict quarantine including social distancing and isolation has affected many aspects of people's lives thereby resulting also in psychological problems (3).

Studies reviewed the psychological impact of quarantine and found negative effects including post-traumatic stress symptoms, confusion and anger (3–5). Having organic disease was an independent risk factor for insomnia, depression, somatization, and obsessive-compulsive symptoms among medical health workers during the epidemic in China (6). In particular, in populations that are more vulnerable a special level of awareness concerning mental health is needed in the current situation to allow for better management of psychiatric symptoms and prevention for suicide (7–9).

The ongoing pandemic is having a drastic impact on all aspects of national healthcare systems including solid organ transplantation services, that represents a field with a high demand of stringent management. Kidney-transplant recipients appear to be particularly at high risk for critical COVID-19 illness (10) and have been reported to have a high early mortality rate (11). From the earliest stages of the COVID-19 pandemic there was a concern that solid organ transplant recipients would be particularly vulnerable to infection and may experience a more severe clinical course (11, 12).

Consequently, it could be hypothesized that the temporary suspension of transplantation procedures as well as the social distancing may result in an increase in psychosocial stress in patient with end-stage liver or end stage kidney disease on transplant waiting lists with resultant higher rates of depression, anxiety, insomnia and harmful drinking in both groups, despite the specific differences between both groups. Depression and anxiety are common triggers for increased alcohol consumption as a form of self-medication in patients who are already at high risk (13, 14). During the waiting list period, liver transplant candidates showed distinct trajectories of anxiety and depression with an increase in symptom level over time (15).

In addiction to uncertainty, pre-transplant depression was significantly associated with a prolonged length of stay in hospital after transplantation, discharge disposition and reduced long-term survival after liver transplantation (16–18).

Social support and functioning is an important variable leading to psychological and social well-being in transplant patients (19) and recognized in coping with stress and health treatment adherence (20, 21). Social distancing and isolation during the coronavirus pandemic may potentiate psychological distress and COVID-19-associated fears.

Based on these risk factors this study aimed to investigate the psychosocial burden of patients on the waiting list for transplantation during the Austrian lockdown in April 2020. We hypothesized that patients on the waiting list for liver or kidney transplantation may report more psychological symptoms (anxiety, depression, somatization) and more sleep disturbances compared to healthy controls. In addition, we predicted that people living with reduced social contacts would show more symptoms compared to people with more social contacts and that participants with an impairment in personality structure, which represents the capacities for self and object recognition, regulation, communication and attachment would report more symptoms compared to healthy controls.

Thus, we conducted a single-institution prospective analysis to address the following questions:

1. Is there a significant difference in symptom load (including depression, anxiety and somatization), depression and sleep quality between patients on the waiting list for liver or kidney transplantation compared to healthy controls during the COVID-19 pandemic?
2. Do patients and controls differ in COVID-19 fears and emotional distress due to social distancing? Is there an association between clinical scales and COVID-19 fears/emotional distress?
3. Are there differences in lifestyle changes (i.e., alcohol consumption, daily structure) in patients compared to controls? Which lifestyle and demographic variables are related to COVID-19-associated fears and symptom load in patients on the transplantation waiting list?
4. Is there an association between personality structure and COVID-19 fears and emotional distress due to the coronavirus pandemic and the resultant socioeconomic restrictions?

## Materials and Methods

Study participants of the patient group were recruited from patients on the waiting list for kidney or liver transplantation at the General, Visceral and Transplant Surgery, Medical University of Graz. Further inclusion criteria were age of 18 years or older and existence of an e-mail account. Informed consent was obtained before each participant completed the online questionnaire. The study measures included demographic variables as well as a standardized psychological test battery described in detail below. The data were acquired via the online-survey platform *LimeSurvey* (www.limesurvey.org). The study was approved by the local ethics committee of the Medical University of Graz (EK Nr: 32-062 ex 19/20). The assessment was carried out in April 2020.

Controls were included from the BIPLONG study (EK Nr: 24–123 ex 11/12) investigating amongst others the psychological impact of COVID-19 pandemic in individuals with psychiatric disorders carried out by the Department of Psychiatry and Psychotherapeutic Medicine Graz.

An online survey was performed from April 9 to April 28, 2020. Patients on the waiting list for transplant at the Medical University of Graz, General, Visceral and Transplant Surgery were contacted via phone and e-mail addresses and invited to participate in the study. All patients on the waiting list for liver or kidney transplantation (*n* = 89) were informed about the study *via* telephone and asked for their participation. Of these, 10 patients refused participation, 15 patients had to be excluded because they could not provide e-mail addresses and 64 patients were agreeable to supplying their e-mail addresses. In total, 35 patients started the survey and 27 patients completed the survey from start to finish and could be included in the statistical analyses.

The following psychometric inventories were assessed online:

### COVID-19 Questionnaire

A COVID-19 questionnaire was created by the Department of Psychiatry and Psychotherapeutic Medicine Graz assessing COVID-19-associated fears as follows:

*On a scale from 0–10, how strongly do you rate your concerns and fears about the coronavirus?*

*On a scale from 0–10, how strongly do you rate your fear of contracting the coronavirus?*

*On a scale from 0–10, how strongly do you rate your fear of infecting others with the coronavirus?*

As all the items showed a highly significant intercorrelation (all *p* < 0.01), a mean index for COVID-19 associated fears (“COVID-19 fears”) was created, showing a Cronbach's alpha of α = 0.79.

Emotional distress due to social distancing was assessed by the following five items on a six-point rating scale “*On a scale from 0–5, social distancing makes me feel lonely/bored/frustrated/hopeless/anxious*.” Out of these significantly intercorrelated items (all *p* < 0.01), a mean index for “*Emotional distress due to social distencing*” was created, showing a Cronbach's alpha of α = 0.81.

### Depressive Symptoms

The Beck Depression Inventory (BDI-2) provides objective data by transforming the symptoms of depression into numerical values. The scale consists of 21 items and has a 4-point Likert type scoring system. The total score ranges from 0 to 63 points. According to the manual, a score below 9 indicates a lack of clinical depression and has been shown to demonstrate an internal consistency with a Cronbach's α ≥ 0.84 and a reliability of *r* ≥ 0.75 (22).

### Global Symptom Load

The Brief Symptom Inventory (BSI-18) (23, 24) measures psychiatric symptoms and global symptom load and is a short version of the Symptom-Checklist (SCL-90-R). The BSI-18 comprises 18 items assessing psychological distress in the last seven days on three subscales (Depression, Anxiety, and Somatization). The Global Severity Index (GSI) is a global measure of psychological distress. The BSI-18 employs a 5-point rating form ranging from 1 (absolutely not) to 5 (very strong). The subscales (total value of each scale is 24) show an internal consistency with a Cronbach's alpha of Somatization α = 0.82, Depression α = 0.87, Anxiety α = 0.84 and GSI α = 0.93 (25).

### Sleep Quality

The Pittsburgh Sleep Quality Index (PSQI) assesses self-reported sleep quality (e.g. sleep duration, onset latency) over the past month. The global score served as the outcome of interest with higher scores indicating poorer sleep quality. Nineteen individual items generate seven “component” scores: subjective sleep quality, sleep latency, sleep duration, habitual sleep efficiency, sleep disturbances, use of sleeping medication and daytime dysfunction. Acceptable measures of internal homogeneity, consistency (test-retest reliability) and validity were obtained with a global PSQI score greater than 5 yielded a diagnostic sensitivity of 89.6% and specificity of 86.5% (kappa = 0.75, *p* < 0.001) in distinguishing good and poor sleepers (26).

### Alcohol Consumption

The AUDIT alcohol consumption questions (AUDIT-C) consists of 10 items, 3 each concerning alcohol consumption or and alcohol addiction, and 4 items that relate to alcohol abuse. A total score is calculated from all questions with possible values between 0 and 40. A score ≥ 8 reveals problematic drinking behavior with reference to alcohol addiction. To compensate for the gender gap, a threshold of 7 is recommended for women and men older than 65 years. The AUDIT-C demonstrated good test-retest reliability (intraclass correlation coefficient for test-retest reliability = 0.91) and satisfactory convergent validity (27).

### Level of Personality Functioning

The short version of the Operationalized Psychodynamic Diagnosis Structure Questionnaire (OPD-SQS) was used to assess personality functioning in wait list patients. The OPD-SQS is a viable screening instrument for supporting clinical decision making in treatment planning and therapy focus. The OPD-SQS consists of 12 items with three subscales (Self-perception, Contact, Relationship). In this study, the sum score was used reaching from 0 (“highest structural level”) to 48 (“lowest structural level”). The internal consistencies range from α = 0.87 to 0.89 (28).

In addition, sociodemographic data including sex, age, education, relationship status, information about the living and job situation, education, subjective changes in physical activity, engagement in activities and hobbies, substance use, medication, diet, use of relaxation methods, the presence of a daily structure and social support, changes in social contact, medication and contact with a mental health professional were assessed.

### Statistical Analyses

The assumption of a normal distribution was tested with the Kolmogorov Smirnov Test (*p* > 0.05). Variables, which were not normal distributed, were analyzed with non-parametric tests. Descriptive results of continuous variables are expressed as means and standard deviations (SD) for Gaussian distributed variables. Differences between patients and controls were tested with *t*-tests (for metric variables). For categorical tests, Chi-squared tests were used and when the expected cell size was <5, an extension of the Fisher's exact test, the Freeman-Halton test was performed. To test the hypotheses, multivariate and univariate analyses of co-variance (MANCOVAs and ANCOVAs controlling for age) and partial correlation analyses (controlling for age) and spearman rank correlations (for single items, e.g., in lifestyle changes) were used. To test differences in single items between the groups, we used non-parametric Mann-Whitney-*U*-tests. Effect sizes are presented as Cohen's partial eta-squared (η*2*). All data met the appropriate assumptions of multivariate normality, linearity and homogeneity of variance. To reduce the type I error from multiple testing, Bonferroni correction was applied for all correlation analyses and the significance niveau was set at *p* < 0.00625 (0.05/8 tests). All analyses were performed using the Statistical Package for Social Sciences (SPSS version 25.0, IBM).

## Results

Table 1 shows demographic characteristic of the sample. Six patients were on the waiting list for liver transplantation and 21 were awaiting kidney transplantation, but they did not significantly differ in relevant sociodemographic or psychological parameters (all *p* > 0.05). In patients, the median AUDIT-C score was 3.3 (SD = 4.32) ranging from 0 to 21 and the median OPD-SQS score was 12.54 (SD = 6.92) ranging from 1 to 29. None of the participants had been tested positive for COVID-19 nor had lived with a positive COVID-19 case at time of testing.

**Table 1:** Demographic data.

	**Patients (*****N*** **= 27)**		**Controls (*****N*** **= 43)**		**Statistics and *P*-value**
	***N***	**%**	***N***	**%**	
**Gender**					
Male Female	13 14	48.1 51.9	11 32	25.6 74.4	χ^2^(1) =3.75, *p* = 0.053
**Age**					
years mean (SD)	48.4 (12.4)		32.2 (7.6)		*T*(68) = 6.09, *P* < 0.001
**Marital status**					
Married, in relationship Single	21 6	77.8 22.2	32 11	74.4 25.6	χ^2^(1) =0.10, *p* =.750
**Education**					
No traditional/formal Compulsory education Apprenticeship A-Levels University bachelors degree University Master's Degree University Doctorate	0 2 15 7 0 2 1	0 7.4 55.6 25.9 0 7.4 3.7	0 0 1 8 8 19 7	0 0 2.3 18.6 18.6 44.2 16.3	*P* < 0.001^a^
**Employment**					
Unemployed, before the pandemic Unemployed, due to the pandemic Short-time work Retired Student Home office Employed	1 1 3 17 1 3 1	3.7 3.7 11.1 63.0 3.7 11.1 3.7	1 0 7 0 7 16 12	2.3 0 16.3 9 16.3 37.2 27.9	*P* < 0.001^a^
**Living situation**					
Alone With partner With partner and children With child/children In a shared residence With parents In a multi-generation household	3 16 4 0 0 3 11	11.1 59.3 14.8 0 0 11.1 3.7	4 19 8 1 3 7 1	9.3 44.2 18.6 2.3 7.0 16.3 2.3	*P* = 0.693^a^
**Inhabitants town of residence**					
<1,000 inhabitants 1,000–4,999 inhabitants 5,000–9,999 inhabitants ≥10,000 inhabitants	4 11 3 9	14.8 40.7 11.1 33.3	1 2 7 33	2.3 4.7 16.3 76.7	*P* < 0.001^a^

a *Comparison with Fisher's exact probability test*.

### Scientific Question 1: Differences in Psychological Symptoms

**Figure 1:**
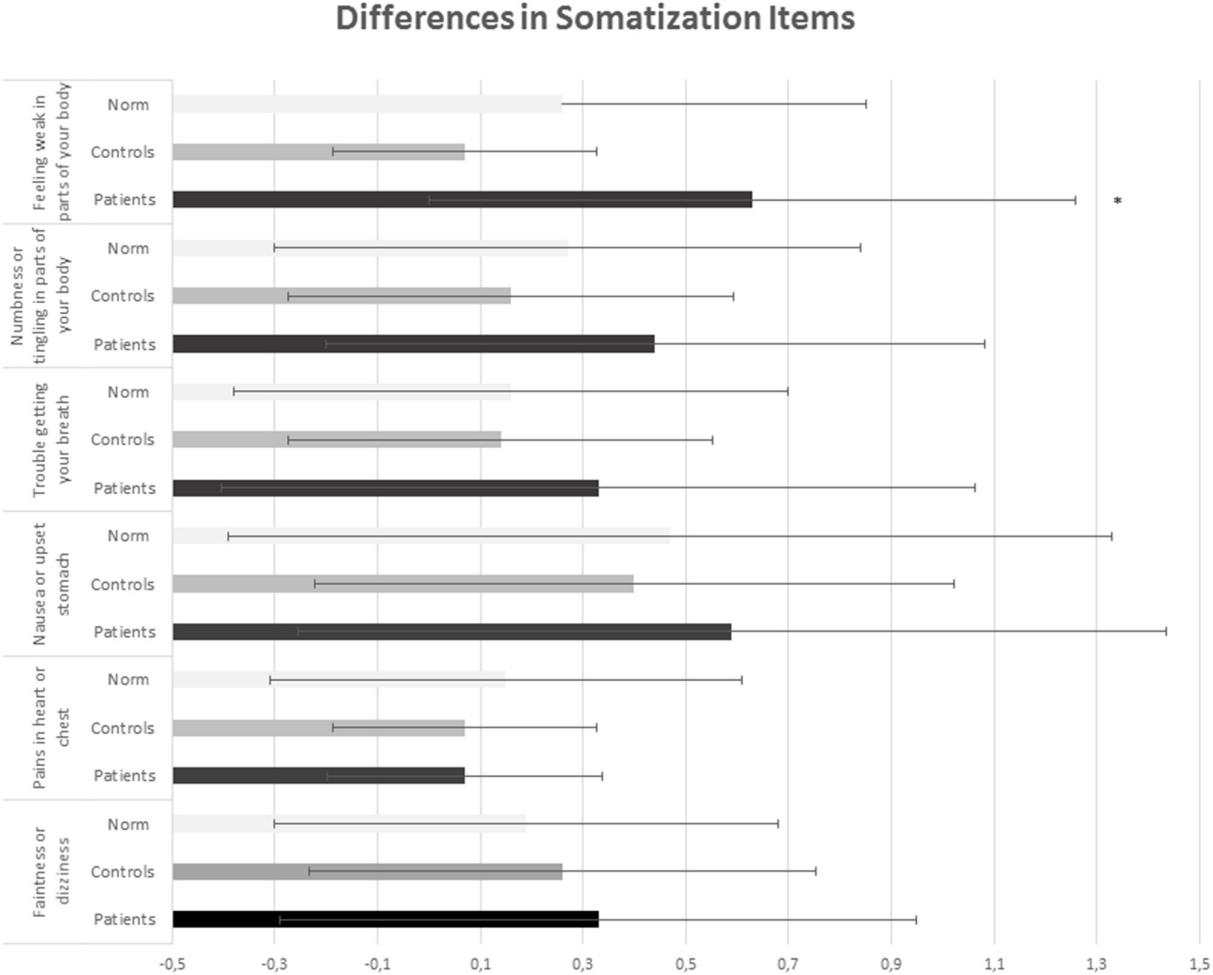
Differences in somatization Items between patients and controls. *Indicates significants.

No MANCOVA differences in BSI-18 subscales between patients and controls were observed (*F*_(3,64)_ = 1.502, *p* = 0.222, η*2* = 0.078). However, the univariate results indicated a difference between patients and controls in the BSI-18 subscale Somatization with higher values in the patient group. In Table 2 means and standard deviations in BSI-18 subscales and BSI-18 GSI in patients and controls are described. Figure 1 shows group differences in the Somatization items.

**Table 2:** Means and standard deviations in BSI subscales, BSI GSI, BDI-2, and PSQI in patients and controls.

	**Group**	**Mean**	**Std.-deviation**	***F***
BSI-18 somatization	Patients	2.41	2.45	4.41^*^
	Controls	1.09	1.56	
BSI-18 depression	Patients	1.11	2.14	0.608
	Controls	1.81	2.10	
BSI-18 anxiety	Patients	1.33	1.50	0.424
	Controls	1.56	1.33	
BSI-18 GSI	Patients	.270	.269	2.44
	Controls	.248	.217	
BDI-2 sum score	Patients	3.33	3.92	0.00
	Controls	3.60	3.00	
PSQI sum score	Patients	3.67	2.43	1.23
	Controls	2.74	2.48	

*N = 70 (27 patients, 43 controls); BSI-18 = Brief Symptom Inventory-18; GSI = Global Severity Index; BDI-2 = Beck Depression Inventory-2; PSQI = The Pittsburgh Sleep Quality Index*.

* *p < 0.05*.

*Significant differences are indicated in bold*.

In BDI-2, neither patients nor controls showed depressive symptomatology (both patients and controls had a mean BDI-2 score below 4) or severe sleeping problems (patients and controls had a PSQI sum score below 4) during the coronavirus pandemic. Accordingly, variance analytic results showed no differences in BDI-2 (*F*_(1,66)_ =.00, *p* = 0.998) nor PSQI sleep components (*F*_(7,54)_ = 1.23, *p* = 0.300, η*2* = 0.137; see Table 2) between patients and controls.

### Scientific Question 2: COVID-19 Fears and Emotional Distress Due to Social Distancing

ANCOVAs showed no differences between patients and controls in the index of emotional distress due to social distancing (*F*_(1/67)_ = 0.39*, p* = 0.533, η2 = 0.006), nor in COVID-19 fears (*F*_(1/67)_ = 1.06, *p* = 0.306, η*2* = 0.016). Testing differences in the three single items of COVID-19 fears using non-parametric tests, we found a tendency to greater fear of infecting others with the coronavirus in the control group than in the patient group (*U* = 373.5, *Z* = −2.52, *p* = 0.012; patients mean score = 3.30, SD = 3.44, controls mean score = 5.12, SD = 2.50).

The partial correlation analysis controlling for age indicated that the index of COVID-19 fears was associated with BSI Somatization in the patient group only (*r* = 0.47, *p* = 0.015; see Table 3), but these result did not survive Bonferroni correction. Additional spearman-rank correlation analyses testing the three single items of COVID-19 fears indicated that Somatization correlated significantly with the fear of contracting the coronavirus (*r* = 0.53, *p* = 0.004) in the patient group. No significant associations were found between COVID fears and BS-18 scales in the control group.

**Table 3:** Correlations between COVID-associated fears and emotional distress due to social distancing with symptom load scales (BSI-18), depressive symptoms (BDI-2), and sleeping quality (PSQI).

	**Group**	**Covid-19 fears**	**Emotional distress due to social distancing**
BSI-18 somatization	Patients	0.47^*^	0.36
	Controls	0.27	0.11
BSI-18 depression	Patients	0.19	0.76^***^
	Controls	0.27	0.56^***^
BSI-18 anxiety	Patients	0.44^*^	0.54^**^
	Controls	0.36^*^	0.61^***^
BSI-18 GSI	Patients	0.47^*^	0.67^***^
	Controls	0.37^*^	0.55^***^
BDI-2	Patients	0.40^*^	0.58^**^
	Controls	0.39^*^	0.58^***^
PSQI	Patients	0.33	0.38
	Controls	0.29	0.35^*^

*Results from partial correlation analysis controlling for age; BSI-18 = Brief Symptom Inventory-18; GSI = Global Severity Index; BDI-2 = Beck Depression Inventory-2; PSQI = The Pittsburgh Sleep Quality Index;*

* *p < 0.05,*

** *p < 0.01,*

*** *p < 0.001;*

*Ssignificant results after Bonferroni correction for multiple comparisons are in bold: threshold of significance: p < 0.00625 (0.05/8 tests)*.

Positive correlations between the index of COVID-19 fears and Anxiety, GSI and BDI-2 scores were observed in both groups (all *p* < 0.05); however these associations were no longer significant after Bonferroni correction (see Table 3). In contrast, partial correlation analyses controlling for age between the index of emotional distress due to social distancing and the psychological scales indicated strong positive correlations in both groups (see Table 3). Emotional distress due to social distancing correlated with Anxiety, Depression and GSI as well as BDI-2 in both groups. Sleeping disorders (PSQI) were related to emotional distress due to social distancing only in controls, but this effect disappeared after Bonferroni correction (see Table 3).

### Scientific Question 3: Subjective Lifestyle Changes

In Table 4, the subjective lifestyle changes due to the pandemic are presented. In controls, 16 out of 43 reported to use relaxation methods such as yoga or progressive muscle relaxation often or sometimes, whereas this was indicated by only 3 patients out of 27. Patients reported to drink less alcohol than healhy controls (14 reported to never drink alcohol vs. 4 controls; χ2(4) = 17.39, *p* = 0.002). Spearman rank correlations showed that in patients, the change of having social contacts (more social contacts during the pandemic) correlated with the mean index of COVID-19 fears (*r* = 0.44, *p* = 0.026); however, this was not significant after Bonferroni correction. There were no associations between the other lifestyle variables (physical activity, engagement in activities and hobbies, change in alcohol consumption, use of relaxation methods, having a daily structure) or demographic variables (education, marital status, employment, living situation, inhabitants) with COVID-19 fears and psychological scales (all *p* > 0.05) in both patients and controls.

**Table 4:** Lifestyle factor changes due to the pandemic.

	**Patients (*****n*** **= 27)**		**Controls (*****n*** **= 43)**		
	***N***	**%**	***N***	**%**	***P*-value^a^**
Change in physical activity More active Less active No change	7 7 13	25.9 25.9 48.1	9 21 13	20.9 48.8 30.2	0.158
**Change in activities or hobbies**					
More engagement Less engagement No change	10 3 14	37.0 11.1 51.9	20 11 12	46.5 25.6 27.9	0.120
**Changes in social contacts**					
More social contacts Less social contacts No change	4 12 11	14.8 44.0 40.7	4 25 14	9.3 58.1 32.6	0.527
**Change in alcohol consumption**					
More alcohol use Less alcohol use No change missing	0 2 11 14	0 7.4 40.7 51.9	9 8 22 4	20.9 18.6 51.2 9.3	0.113
**Daily structure**					
Yes Partially No	13 13 1	48.1 48.1 3.7	37 18 1	52.9 41.9 2.3	0.236
**Use of relaxation methods**					
Often/sometimes Seldom Never	3 6 18	11.1 22.2 66.7	16 10 17	37.3 23.3 39.5	**0.032**

a *all comparisons with Fisher's exact probability test. Significant values (p < 0.05) in bold*.

### Scientific Question 4: Personality Structure and COVID-19 Fears

The partial correlation analysis showed no relationship between OPD-SQS and COVID-19 fears/emotional distress due to social distancing in patients on the transplant waiting list (all *p* > 0.05).

## Discussion

Our online survey study aimed at assessing the impact of the COVID-19 pandemic including restrictions in the Austrian lockdown on psychosocial functioning of patients on the waiting list for liver or kidney transplantation. The results demonstrate that during the COVID-19 pandemic transplant waiting list patients show increased somatization symptoms compared with controls. The elevated somatization scores were associated with COVID-19 fears in transplant waiting list patients; however this association was weak. Depressive symptoms and anxiety were related to emotional distress due to social distancing in both, patients and controls.

Somatization refers to psychological stress caused by the perception of physical dysfunctions. The items focus on body symptoms with strong autonomous mediation (29–31). This subjective representation may determine a patient's coping behavior. Somatization may be viewed as a primary driver for higher perception, symptom reporting, health care use, symptom persistence, and negative treatment outcome (32–34).

In addition, Somatization was associated with COVID-19 fears in patients, especially, the fear of contracting the coronavirus. It is very important to understand the psychological basis of anxiety and fears associated with the pandemic and to counter them by finding evidence-based ways of addressing these issues for future outbreaks of infection or crisis (35). Severe misregistrations of autonomous regulation can impaire the patients' sensitive and psychoneuroimmunological state and consequently transplantation outcome.

Depression and Anxiety were highly associated with emotional distress due to social distancing in both groups confirming the negative effects of lockwdown restrictions and social distancing on psychological well-being. Interestingly, there was no difference in depressive symptoms between patients and controls. This might be because of the lacking ability of the BDI-2 to differentiate in non-psychiatric samples, due to the small sample size or other reasons like the possibility that the healthy population was affected to the same extent as the patients or that possible symptoms related to the crisis only present after some delay. In other studies, pre-transplant depression was significantly associated with greater length of stay in hospital post-transplant and mortality (16). However, depressive symptoms measured with the BSI-18 correlated positively with emotional distress due to social distancing. The long-term effects of the COVID-19 pandemic on depressive symptoms has to be evaluated in follow-up investigations.

A study from China reported more posttraumatic stress symptoms in the domains of re-experiencing, negative alterations in cognition or mood and hyper-arousal during outbreak in hardest hit areas (36). The results of this study did not show differences in emotional distress due to social distancing, but there was a difference in COVID-19 fears indicating more fear of infecting others with the coronavirus in the control group compared with the patient group. This may be explained by a difference in the awareness of illness, subjective meaning and responsibility to others between patients and controls during the COVID-19 pandemic. There were no significant differences in lifestyle changes due to the pandemic between patients and controls; however, in the patient group, having more social contacts was related to COVID-19 fears.

We acknowledge several limitations to our study, not least of all the cross-sectional design. The sample was very small and in the accruing sample not every potential confounding variable could be included as a co-variable into the analyses. We could not present separately the data for liver and kidney transplantation waiting list because of the unequal sample size; however pre-analysis showed no differences in psychological scales between the two groups. From the 64 patients who provided email addresses only 27 could be finally analyzed. There were 29 patients, who apparently agreed to provide email but did not start the survey, further 8 patients started but not completed the study questionnaire. The reason for drop-out may be due to the length of the selected measurements with about 20–30 min. Follow-up investigations should use a shortened version of the survey. Another potential bias could be that patients without an email address could not answer the survey. Thus, there was a shift to younger participants especially in the control group. The controls were younger median age and higher educational status; as educational status was not related to the dependent variables, we controlled only for age in the analyses. Our results are also limited by the absence of biological data, which might reveal underlying vulnerability factors. It would need a longitudinal study, to identify changes from a baseline (before COVID-19) and other points of measurement during and post pandemic. However, the aim here was to highlight the impact of this unprecedented world-wide condition on the mental health of this especially vulnerable population for the first time. In this regard, the results lay a foundation for a follow-up study and provide indications to better support of transplant patients in a crisis.

In view of the crisis, data clearly show that there is an urge for multi-disciplinary research in the field of mental health and for the establishment of prevention strategies for psychological COVID-19 consequences (37, 38). Last year, Qiu et al. suggested future interventions such as focusing more attention on vulnerable groups and developing nationwide strategies to plan and coordinate psychological first aid during major disasters using telemedicine (3). Those vulnerable groups which are paying the highest price for the COVID-19 crisis are the elderly and individuals with chronic long-term health conditions (39). Amario et al. give early insights into an urgent need of continuity in the national and regional health care systems including the need of regular outpatient visits and the expansion of telemental health services. In addition, a crisis prevention and intervention plan should be built to reduce psychological distress and prevent mental health problems (3, 40).

## Conclusion

Patients on the waiting list for a transplantation show more somatization symptoms in the COVID-19 pandemic compared to controls. Patients with a higher vulnerability and an impaired self-awareness need more information about their illness, about the possible changes and fears associated with the spread of an infectious disease, their medication and treatment strategies for stress reduction in addition to receiving more information about how to protect themselves from being infected with SARS-CoV2.

## Data Availability Statement

The original contributions presented in the study are included in the article/supplementary material, further inquiries can be directed to the corresponding author/s.

## Ethics Statement

The study was conducted ethically in accordance with the World Medical Association Declaration of Helsinky. All study participants have given their written incformed consent and the sudy protocol was approved by the local institutes committee on human research of the Medical University Graz (EK Nr: 32-062 ex 19/20). The patients/participants provided their written informed consent to participate in this study.

## Author Contributions

JW-S: has designed the study, written the first draft, was responsible for the study conception, for patient recruitment, testing, coordination, and publication of data. ND: was involved in the conception of the study, supervised and guided through the whole process of analysis and publication. SB: was responsible for study conception and design, coordination, funding and drafting/publication of data. MR: managed patient schedules and appointments and supervised the testing. RP: was responsible for the scientific parameters in this project and the formal criteria. CF: was involved in the conception of the study and did revision for important intellectual content. SM: was involved in the conception of the study (selection of questionnaires) and did revision for important intellectual content of the paper as well as she supervised our work during all parts of the study. ML: was involved in the statistical analyses and the preparation of the manuscript. FM: was responsible for the scientific work up, helped with interpretation of the data and did revision for important intellectual content of the paper. NF: was responsible for the clinical, scientific work up, helped with interpretation of the data. JK: was responsible for the clinical and scientific work. ER: supervised the whole study procedure and did revision for important intellectual content. MB: was responsible for proof reading and revising the manuscript as a native speaker. HM: revised the manuscript and did revision for important intellectual content. DK: helped with data interpretation and revised the manuscript. All authors edited and approved the final manuscript.

## Conflict of Interest

The authors declare that the research was conducted in the absence of any commercial or financial relationships that could be construed as a potential conflict of interest.

## Abbreviations

ANCOVA: analysis of co-variance
AUDIT-C: AUDIT alcohol consumption questions
BDI-2: the beck depression inventory
BSI 18: brief symptom inventory-18
COVID-19: coronavirus disease 2019
GSI: global severity index
MANCOVA: multivariate analysis of co-variance
OPD-SQS: operationalized psychodynamic diagnosis structure questionnaire
PSQI: pittsburgh sleep quality index
SARS: severe acute respiratory syndrome coronavirus 2
SCL-90-R: symptom-checklist-90-standard
WHO: World Health Organization.
